# Propolis ethanolic extract has double-face *in vitro* effect on the planktonic growth and biofilm formation of some commercial probiotics

**DOI:** 10.1016/j.sjbs.2020.11.047

**Published:** 2020-11-17

**Authors:** Ibrahim Alfarrayeh, Csaba Fekete, Zoltán Gazdag, Gábor Papp

**Affiliations:** Department of General and Environmental Microbiology, University of Pécs, Faculty of Sciences, Pécs, Hungary

**Keywords:** Dysbiosis, Propolis, Probiotic, Biofilm, Antimicrobial, Food preservative, PEE, Propolis Ethanolic Extract, MIC, Minimal Inhibitory Concentration, PBS, Phosphate-Buffered Saline

## Abstract

This study investigated the *in vitro* effect of propolis ethanolic extract (PEE) on planktonic growth and biofilm forming abilities of five commercial probiotics (Enterol, Protexin, Normaflore, BioGaia and Linex). Broth microdilution method was used to investigate the susceptibility of the microbes of five commercial probiotics to PEE. Crystal violet assay was used for the quantitative assessment of biofilm formation and mature biofilm eradication tests. Effect of PEE on autoaggregation ability and swarming motility of Normaflore microbes was determined. Planktonic forms of probiotics showed varied susceptibilities with minimal inhibitory concentration values in the range of 100–800 µg/mL of PEE. However, low PEE concentrations significantly enhanced the planktonic growth of Linex and BioGaia microbes. Biofilm studies revealed that Enterol and Protexin were non-biofilm formers, while BioGaia, Linex and Normaflore showed weak biofilms, which were inhibited by 12.5, 25, and 800 µg/mL of PEE, respectively. PEE revealed double-face effect on the biofilms of Normaflore and Linex, which were enhanced at low concentrations of PEE and inhibited at higher concentrations. Interestingly, Normaflore biofilms were shifted from weak to strong biofilms at low PEE concentrations (12.5, 25, and 50 µg/mL). In conclusion, PEE has strain dependent controversial effects on the planktonic growth and biofilm forming ability of the tested probiotics, although high concentrations have inhibitory effect on all of them, low concentrations may have strain dependent prebiotic effect.

## 1 Introduction

The gastrointestinal tract is colonized by large number of microorganisms (Lin and Zhang, 2017) which have several health benefits for the host, such as the improvement of intestinal health, harvesting energy, competitive exclusion and antimicrobial activity against pathogens and immune modulation (Thursby and Juge, 2017). Any alterations in the gut microbiota due to environmental factors, including diet, toxins, antibiotic therapies and pathogens can result in a condition known as dysbiosis (Carding et al., 2015). Dysbiosis may develop as temporary or chronic clinical symptoms, or it could be asymptomatic but may increase susceptibility for many diseases, including intestinal, metabolic and brain disorders (Blumstein et al., 2014). This problem can be solved by using probiotics, which have the ability to recolonize the gut, improve its normal microbiota and enhance overall health (Gill and Guarner, 2004, Puebla-Barragan and Reid, 2019). Probiotics are becoming more and more used in veterinary and human medicine (Puebla-Barragan and Reid, 2019, Weese, 2003), and they are commercially available as dietary supplements under several brand names in the markets. Probiotics can grow in the gut in two forms: either freely swimming planktonic cells or as biofilms attached to the intestinal mucosa. Biofilm form of growth of probiotics is considered an advantageous property, since it could resist the environmental conditions, support longer persistence in the gut of the host and prevent colonization by pathogenic microorganisms (Salas-Jara et al., 2016).

Propolis is one of the most important honeybee products, it has been reported to have a wide range of biological activities (Mello and Hubinger, 2012). It is prepared by honeybees as a resinous material to fill the cracks, smooth walls, and to maintain humidity and temperature stability in the colony throughout the year. Propolis as raw material consists of 50% plant resins, 30% waxes, 10% essential and aromatic oils, 5% pollens and 5% other organic substances. It is usually prepared from resinous secretions of poplars, conifers, birch, pine, alder, willow and palm (Bankova et al., 2000). Propolis is used in traditional and modern medicine for the prevention and curing of colds, wounds and ulcers, rheumatism, sprains, heart disease and diabetes (Huang et al., 2014). It has varied biological properties such as anti-inflammatory (Wang et al., 2013), antimicrobial, antioxidant, antitumor (Bankova et al., 2000, Huang et al., 2014), antiulcer and anti-HIV activities (Ito et al., 2001). Its antioxidant and antimicrobial properties provide scope for use in food technology as food preservative. One of the most important advantages is that, unlike the other preservatives, its residues may have a generally favorable effect on the health (Bahtiti, 2013).

The chemical profile of propolis is very complex, and more than 300 constituents have been characterized (Ahangari et al., 2018, Ristivojević et al., 2015). The greatest part of its biological activities can be attributed to the flavonoids, which are extensively present in propolis (Dalben-Dota et al., 2010, Saddiq and Danial, 2014). However, many studies mentioned that the varied biological activities might be due to synergistic action of its components (Ahangari et al., 2018, Bueno-Silva et al., 2013).

Propolis has been reported by many studies to have antimicrobial properties against pathogens (Boyanova et al., 2005, Stepanović et al., 2003, Uzel et al., 2005), but less information is available about the effects that natural antimicrobial agents, like propolis, could have on the normal gut microbiota and probiotic microorganisms consumed for their assumed benefits. This study was conducted to investigate the *in vitro* effect of treatment with propolis ethanolic extract (PEE) alongside probiotics on their planktonic growth and biofilm forming abilities. The tested probiotic products were selected to represent the most common strains that have probiotic properties including fungal and bacterial strains. Five probiotic products were used in this study, three of them contain single species, while the others contain more than one species.

## 2 Materials and methods

### 2.1 Preparation of propolis extract

Propolis sample was collected from a local beekeeper in Pécs/Hungary. It was extracted with ethanol 80% (v/v) in water bath, at 70 °C, for 30 min and then filtered to obtain its ethanolic extract (Alencar et al., 2007). The concentration of the stock solution was set to 222.2 mg/ml.

### 2.2 Test probiotics

Five commercial probiotics were purchased from a pharmacy in Hungary and used in this study: Normaflore® (*Bacillus clausii*), Enterol® (*Saccharomyces boulardii* CNCM I-745), BioGaia® (*Lactobacillus reuteri* DSM 17938), Linex® (*Lactobacillus acidophilus* LA-5 and *Bifidobacterium animalis* subsp. *Lactis* BB-12) and Protexin® (*Lactobacillus paracasei, Lactobacillus rhamnosus, Lactobacillus acidophilus, Lactobacillus bulgaricus, Bifidobacterium breve, Bifidobacterium infantis* and *Streptococcus thermophiles*).

### 2.3 Culturing media and growth conditions

All probiotics were grown in de Man, Rogosa and Sharpe (MRS) broth (Sigma-Aldrich, Switzerland) except Enterol microbes which were grown in YEPD broth (1% yeast extract, 2% peptone, and 2% glucose in distilled water, pH 6.8). The microbes of BioGaia, Linex and Protexin were grown in anaerobic atmosphere at 37 °C for 24 h in GasPak anaerobic system using AnaeroGen™ sacs (Sigma-Aldrich, Japan). Normaflore and Enterol microbes were grown aerobically at 37 °C for 24 h. All stationary-phase cultures of probiotics were prepared according to their growth curves.

### 2.4 Antimicrobial susceptibility testing

Determination of minimal inhibitory concentration (MIC) by the broth microdilution method was performed based on the recommended protocol of the National Committee for Clinical Laboratory Standard Institute (CLSI, 2012, CLSI, 2007). In short, a standardized initial inoculum (0.5 McFarland) was used for all experiments. The tests were performed in sterile, flat-bottom 96-well microplates (Costar®, USA). Equal volumes of cell suspension and PEE solution were dispensed into the wells to get final concentration ranging from 12.5 to 800 µg/ml. For Linex, BioGaia and Protexin, treatment with glutathione (0–100 µg/ml) was also applied to confirm the effect of antioxidants on the growth of probiotics that contain anaerobic and/or microaerophilic bacteria. For each experiment, negative controls (media and cell suspension without PEE addition) and blanks (media with PEE) were included. The plates were placed in an incubator at 37 °C, and after incubation for 24 h, the optical density (OD) at wave length 600 nm was measured using plate reader (Multiskan Ex, Thermo). The MIC_80_ of PEE was defined as the lowest concentration with a growth reduction (80%) when compared to that of negative control.

### 2.5 Biofilm forming ability assay

Biofilm formation was assayed by the ability of cells to adhere to the wells of a 96-well tissue culture microplate (Sarstedt, Germany). Biofilm formation assay was done as described by Stepanović and co-workers (Stepanović et al., 2007). To inoculate the biofilm forming ability assay microplates, 0.5 McFarland standard equivalent cell number was applied to prepare stationary-phase probiotic culture. Stationary-phase culture was vortexed and thereafter diluted 1:100 using RPMI-1640 medium (Sigma-Aldrich, Saint Louis, USA). The stock solution of PEE was used to prepare series of 2-fold dilutions. Equal volumes of these dilutions were added to equal volumes of the diluted cell suspensions to get final concentrations ranging from 12.5 to 800 µg/ml. Negative controls and blanks were included in each experiment. Solvent concentration was always kept as 1%. The microplates were incubated at 37 °C for 24 h, afterwards the liquid part was removed, and the remaining biofilms were repeatedly washed with Phosphate-Buffered Saline (PBS) (pH 7.4). The biofilms were fixed with 2% formalin-PBS, and stained with 0.13% crystal violet for 20 min at room temperature. The unincorporated crystal violet was removed and the wells were washed thoroughly and repeatedly with PBS buffer. Biofilm formation was quantified by adding 1% SDS solution to each well to solubilize the stain overnight, and the OD of the solution was measured at 600 nm using plate reader. The cut-off values of optical density (OD_c_) were established according to the formula (Stepanović et al., 2007):

$${\text{OD}}_{\text{c}}=\text{average}\;{\text{OD}}_{\text{blank}}+\left(3\times \text{SD}\;\text{of}\;{\text{OD}}_{\text{blank}}\right)$$

where OD_c_ is the cut-off value of optical density, average OD_blank_ is the average of three optical density measurements of blank (media with the proper concentration of PEE), and SD is the standard deviation of three measurements of OD_blank_. Final OD values of the tested probiotics (OD_f_) were generated as the average of three measurements. Based upon the OD_f_ values, probiotics were classified according to Stepanović and co-workers (Stepanović et al., 2007) with some modifications into three categories: non biofilm formers (OD_f_ ≤ OD_c_), weak biofilm formers (OD_c_ < OD_f_ ≤ 2OD_c_) and strong biofilm formers (OD_f_ > 2OD_c_).

### 2.6 Biofilm eradication assay

For the inoculation of the assay microplates, stationary-phase culture was prepared by applying 0.5 McFarland standard equivalent cell number and thereafter diluted 1:100 using RPMI medium. Microplates containing diluted probiotic cell suspension were incubated at 37 °C for 24 h. After the biofilm maturation, PEE treatment was applied. Accordingly, the original RPMI culture was discarded, and replaced with PEE-containing RPMI medium with concentrations ranging from 12.5 to 800 µg/ml. Negative controls and blanks were included in each experiment. After 24 h of incubation at 37 °C, the growth of free-living cells was estimated by measuring the OD of the liquid part of the media at 600 nm, and the remaining biofilms were washed, fixed, stained and estimated as mentioned in the previous section.

### 2.7 Autoaggregation assay

Autoaggregation ability was investigated as described by Jeon and co-workers (Jeon et al., 2017). Briefly, stationary-phase cultured cells were collected by centrifugation (4000*g*, 5 min), washed twice with PBS, and re-suspended in a final cell density equivalent to 1 McFarland standard in PEE-containing PBS in concentration range from 12.5 to 50 µg/ml. Negative controls and blanks were included in each experiment. OD at 600 nm was measured immediately at zero time and after 24 h of incubation at 37 °C, and the percentage of autoaggregation was calculated as follows:

Autoaggregation (%) = (1 – (A_24_/ A_0_)) × 100 where A_0_ and A_24_ represented the optical density at zero time and at 24 h, respectively.

### 2.8 Swarming motility assay

Swarming motility assay was done as described by O’May and co-workers (O’May et al., 2012). In short, basic MRS broth supplemented with 5 g/L of D-glucose and solidified with 0.5% agar (Fluka, Switzerland) were used to prepare swarm agar plates. PEE was added to the swarm agar to get final concentrations ranging from 12.5 to 50 µg/ml. Swarm agar plates were inoculated with 5 µl aliquot of probiotics broth culture. Negative controls were included in all of the experiments. After 24 h of incubation at 37 °C, the diameters of the swarming motility zones were measured and expressed as percentage of the negative control.

### 2.9 Statistical analysis

All experiments were done in triplicates, and data were expressed as mean value ± standard deviation. Statistical analysis was performed either with one-sample or two-sample t-tests using Past 3.21 software.

## 3 Results

### 3.1 Effect of PEE on free-living probiotic cells

**Fig. 1:**
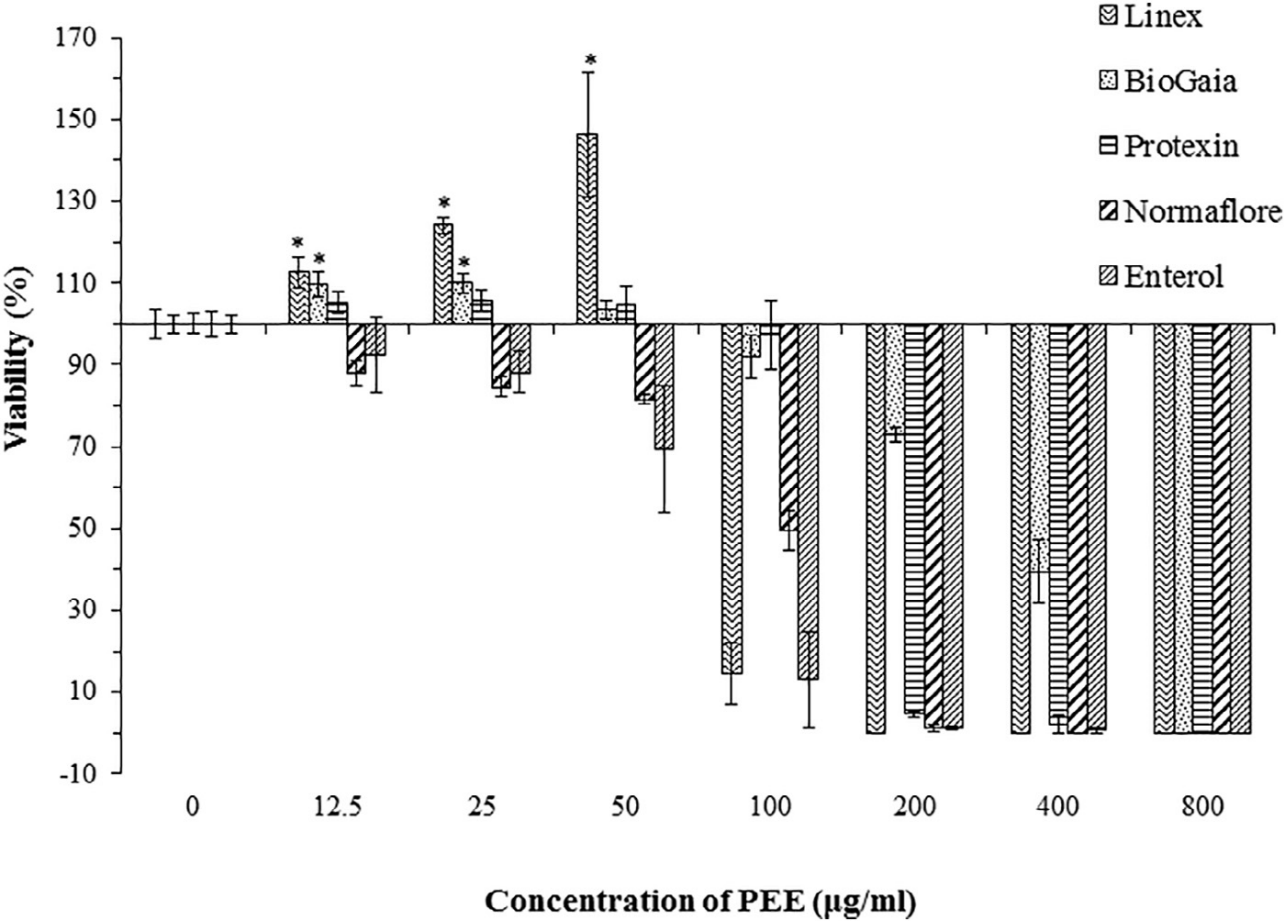
Viability of planktonic form of probiotics in the presence of different concentrations of PEE. Data are shown as the mean ± SD from three independent experiments. *p < 0.05 indicates significant increment of the viability compared to the negative control (0 µg/ml).

To collect basic information about the effect of PEE on the viability of free-living probiotic cells in five different probiotic products, the susceptibility test was conducted. The results revealed that PEE has different effects on the viabilities of the different probiotics (Fig. 1). Due to the known antimicrobial activity of PEE, it has the ability to reduce the free-living form of growth in each case. However, lower concentrations of PEE improved the viability of Linex, BioGaia and Protexin microbes which are mostly containing anaerobic and/or microaerophilic bacteria (Fig. 1). Similarly, the viability of those microbes were also improved when glutathione treatment was applied (Data are not shown).

Based on the performed experiment, MIC_80_ values have been determined. As Fig. 1 demonstrates, different probiotics have varied MIC_80_ values in the range of 100–800 µg/ml. The lowest MIC_80_ value was found for Linex and Enterol (100 µg/ml), while it was doubled for Protexin and Normaflore (200 µg/ml). However, BioGaia, which contains *L. reuteri* DSM 17938, has the highest MIC_80_ value (800 µg/ml), and this might be due to the antibiotic-producing properties of this strain.

### 3.2 Effect of PEE on biofilm forming ability of the probiotics

The importance of biofilm forming ability related to probiotics is unquestionable. Each applied probiotic was tested, and the results revealed that Protexin and Enterol microbes were non-biofilm formers under the applied conditions. On the other hand, the microbes of BioGaia, Linex and Normaflore can form weak biofilms (Table 1).

**Table 1:** Effect of PEE on the biofilm forming abilities of probiotics.

PEE Conc. (µg/ml)	Protexin			Enterol			BioGaia			Linex			Normaflore		
	OD_f_	OD_c_	Biofilm	OD_f_	OD_c_	Biofilm	OD_f_	OD_c_	Biofilm	OD_f_	OD_c_	Biofilm	OD_f_	OD_c_	Biofilm
0	0.086	0.094	NB	0.073	0.095	NB	0.080	0.079	WB	0.130	0.109	WB	0.129	0.067	WB
12.5	0.131	0.172	NB	0.100	0.149	NB	0.128	0.142	NB	0.224	0.147	WB	0.333	0.123	SB
25	0.145	0.200	NB	0.109	0.143	NB	0.128	0.161	NB	0.153	0.168	NB	0.398	0.156	SB
50	0.164	0.203	NB	0.124	0.170	NB	0.144	0.183	NB	0.152	0.197	NB	0.398	0.191	SB
100	0.189	0.247	NB	0.140	0.174	NB	0.166	0.203	NB	0.169	0.217	NB	0.311	0.267	WB
200	0.218	0.310	NB	0.173	0.215	NB	0.223	0.267	NB	0.212	0.251	NB	0.321	0.300	WB
400	0.198	0.228	NB	0.176	0.176	NB	0.218	0.221	NB	0.224	0.313	NB	0.298	0.252	WB
800	0.168	0.234	NB	0.164	0.186	NB	0.172	0.240	NB	0.175	0.254	NB	0.212	0.236	NB

NB: no biofilm (OD_f_ ≤ OD_c_), WB: weak biofilm (OD_c_ < OD_f_ ≤ 2OD_c_), SB: strong biofilm (OD_f_ > 2OD_c_).

The weak biofilm forming probiotics have various responses to the PEE treatments. BioGaia microbes were highly sensitive to the PEE treatment, the lowest concentration of PEE (12.5 µg/ml) was enough to inhibit its biofilm forming ability. However, the same concentration of PEE has slight positive effect on the biofilm forming ability of Linex microbes, but it is still falls to the weak biofilm category. Interestingly, the biofilm of Normaflore microbes showed unique property not only to tolerate higher concentrations of PEE, but moreover, it has been enhanced and shifted from weak to strong biofilm at 12.5, 25 and 50 µg/ml concentrations (Table 1).

### 3.3 Effect of PEE on mature biofilms of Normaflore

**Fig. 2:**
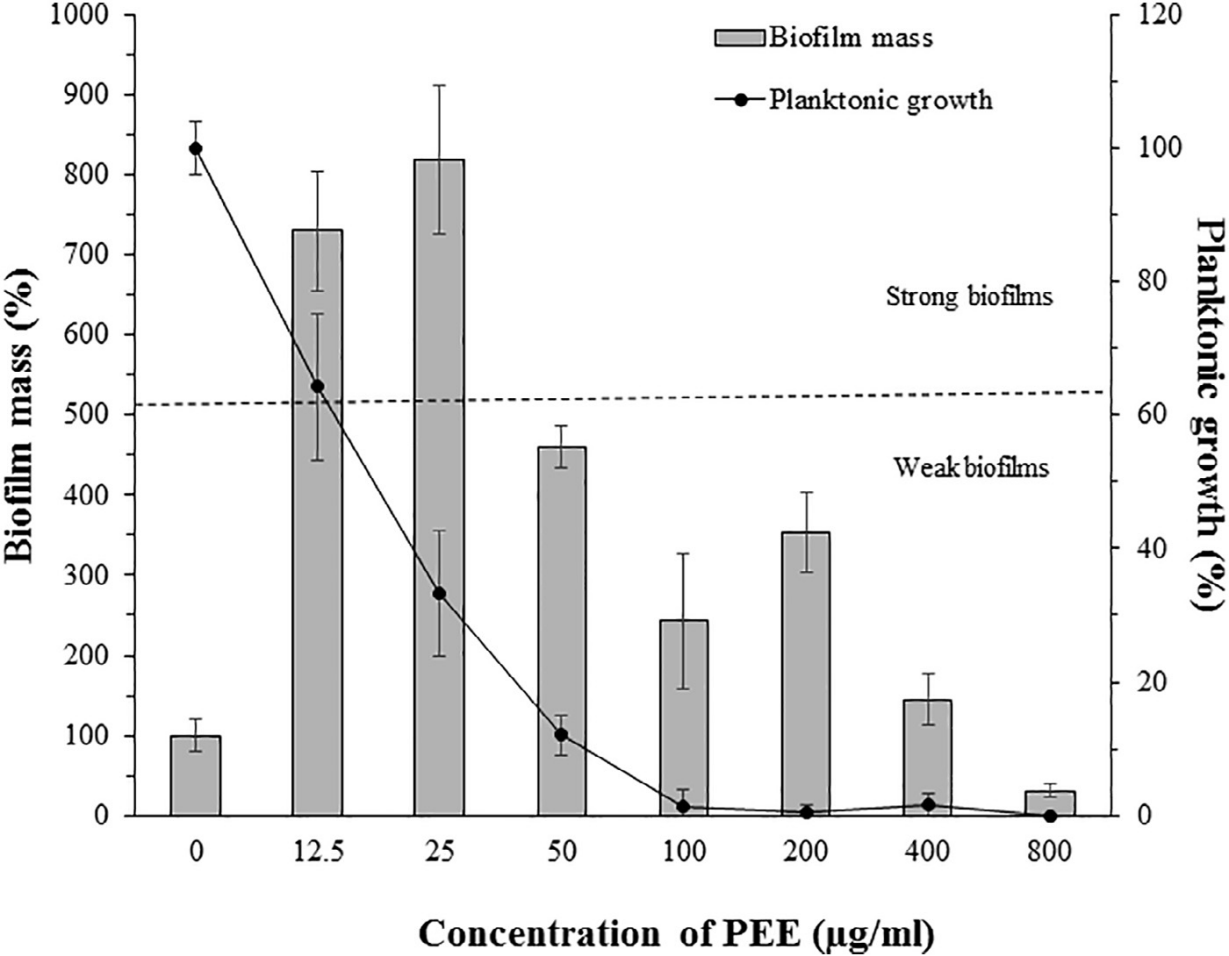
Effect of different concentrations of PEE on mature biofilms and planktonic cells of Normaflore microbes. Dashed line indicates the threshold of strong and weak biofilms. Data are shown as the mean ± SD from three independent experiments.

To get more inside to the unique and interesting response of Normaflore microbes to the PEE treatment, the effect of PEE on their mature biofilms was investigated. Application of different concentrations of PEE revealed that the mass of the mature biofilm was improved up to 400 µg/ml. Whereas biofilm eradication was observed at 800 µg/ml and the biofilm mass was reduced about 70% of the control (Fig. 2). Interestingly, the mature biofilms of Normaflore microbes were shifted from weak to strong at low concentrations of PEE (12.5 and 25 µg/ml). With respect to planktonic cells which were found in the suspension above the biofilm, PEE revealed a dose-dependent inhibitory effect on their growth (Fig. 2).

### 3.4 Effect of PEE on autoaggregation in Normaflore

**Fig. 3:**
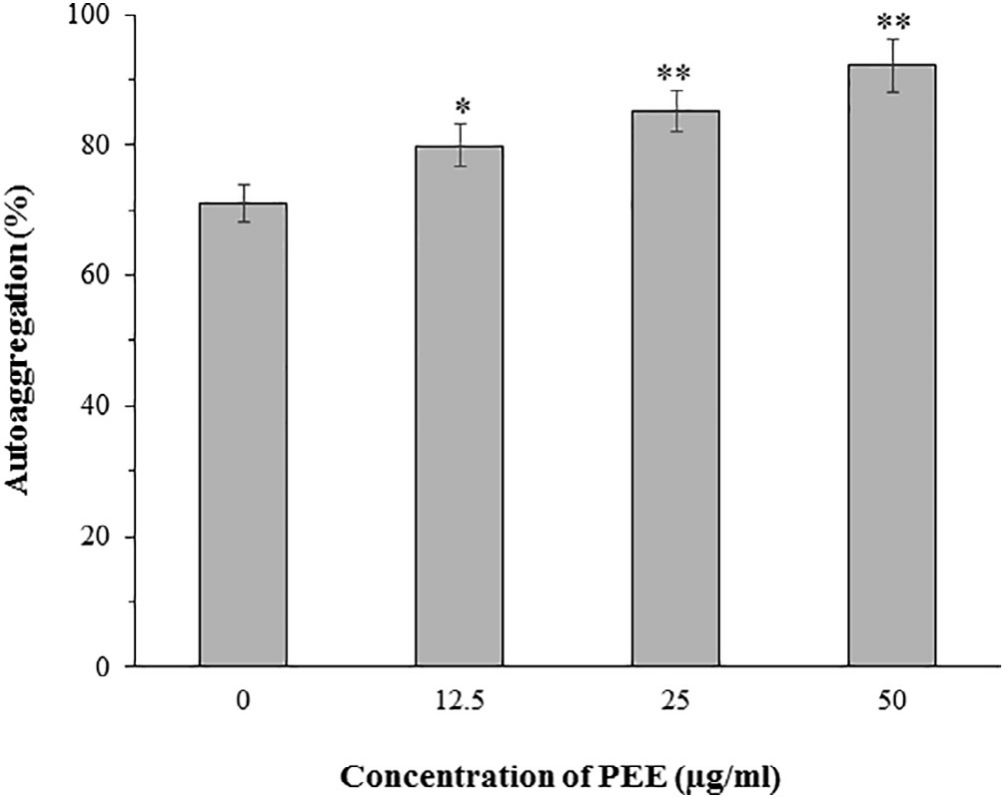
Autoaggregation ability of Normaflore microbes after 24 h incubation in the presence of PEE. Data are shown as the mean ± SD from three independent experiments. *p < 0.05 and **p < 0.01 indicate significant differences compared to the negative control (0 µg/ml).

Autoaggregation is the process by which bacterial cells belonging to the same bacterial strain recognize each other and form multicellular clumps (Trunk et al., 2018). Autoaggregation is known to be positively correlated to biofilm formation ability (Sorroche et al., 2012). The results of the autoaggregation experiment revealed that PEE has significant stimulatory effect on the autoaggregation ability of Normaflore microbes (Fig. 3). After 24 h of incubation, the autoaggregation rate at 12.5, 25 and 50 µg/ml of PEE was about 9, 14 and 21% higher than the negative control (0 µg/ml), respectively. Concentrations above 50 µg/ml were excluded from this experiment because more than 50% of the cells were unviable at these concentrations (Fig. 1).

### 3.5 Effect of PEE on swarming motility in Normaflore

**Fig. 4:**
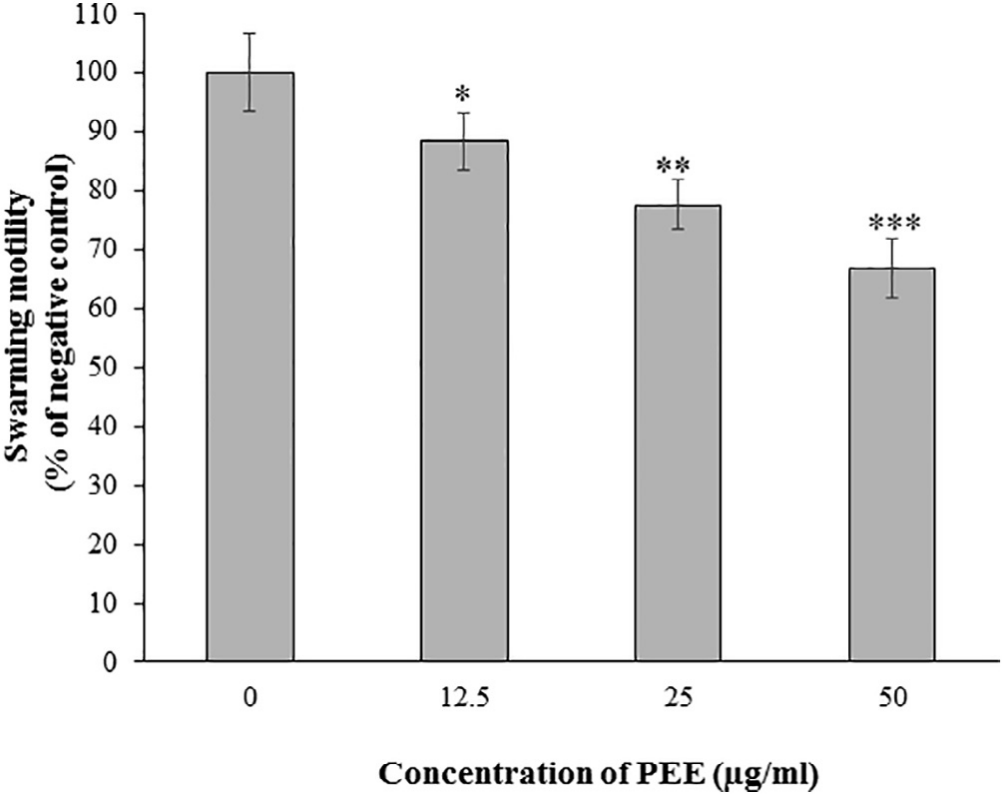
Swarming motility of Normaflore microbes after 24 h incubation with PEE. Data are shown as the mean ± SD from three independent experiments. *p < 0.05, **p < 0.01 and ***p < 0.001 indicate significant differences compared to the negative control (0 µg/ml).

Swarming motility is the rapid and coordinated translocation of a bacterial population on a surface powered by rotating flagella (Kearns, 2010). The findings of this test showed that PEE has significant inhibitory effect on the swarming motility of Normaflore microbes compared to the untreated group (Fig. 4). The rate of swarming motility decreased about 12, 22 and 33% when treated with 12.5, 25 and 50 µg/ml of PEE, respectively.

## 4 Discussion

Recent studies revealed the importance of probiotics for the treatment of dysbiosis after gastrointestinal infections, antibiotics treatment or complementary therapies with natural antimicrobial substances (Ducatelle et al., 2015, McFarland, 2014, Wischmeyer et al., 2016). It is worth to mention that this is the first study that investigates the biofilm formation in commercial forms of probiotics, and evaluate the *in vitro* effects of PEE on their planktonic growth and biofilm forming abilities. Five commercial probiotics were used in this study to estimate their viability and biofilm forming ability with and without PEE treatment. The importance of this study comes from the fact that propolis has antimicrobial properties (Boyanova et al., 2005, Stepanović et al., 2003, Uzel et al., 2005), and this is not only against the pathogenic bacteria, but it might have adverse effects on the growth of the intestinal microbiota and probiotic microorganisms ingested for their benefits (Haddadin et al., 2008). The antimicrobial properties of PEE are attributable to the presence of phenolic compounds, terpenes, caffeic, ferulic and coumaric acids, esters, and flavonoids (Al-Waili, 2018, Inui et al., 2014, Veiga et al., 2017).

The findings of this study revealed that PEE has strain and concentration dependent inhibitory effect on the tested probiotics. However, low concentrations of PEE enhanced the growth of the probiotics that contain anaerobic and/or facultative anaerobic bacteria (Linex, BioGaia and Protexin). This prebiotic effect may be attributable to the antioxidant activity of PEE due to its high total flavonoid and polyphenol contents (Buratti et al., 2007, Laskar et al., 2010, Mohammadzadeh et al., 2007, Naima Benchikha et al., 2014). It has been reported previously that antioxidants can help the growth of anaerobic bacteria (La Scola et al., 2014). This suggestion is supported by the results obtained from the treatment with low concentrations of the standard antioxidant glutathione, that similarly showed the improvement of the growth of the aforementioned probiotics.

BioGaia microbe (*L. reuteri* DSM 17938) has the highest ability to survive and tolerate high concentrations of PEE giving an advantage for BioGaia over the other probiotics. The PEE resistance of *L. reuteri* may be due to its antibiotic-producing ability. *L. reuteri* has been shown to produce the antimicrobial compound reuterin that has antibacterial activity against many bacterial species (Talarico et al., 1988). Although *L. reuteri* itself is less susceptible to reuterin than other bacteria, it can accumulate this compound and generate oxidative stress in their cells (Salminen et al., 2004). The production of reactive oxygen species (ROS) via the binding of reuterin with free thiol groups of proteins and small molecules can result in an enhanced imbalance in cellular redox status (Engels et al., 2016). Although the presence of ROS can limit the growth ability of *L. reuteri*, it is supposed that administration of low concentrations of PEE can act as ROS scavenger enhancing its resistance and cell proliferation. Whereas, higher concentrations of PEE lead to decreased cell survival due to the suppressive antimicrobial effect of its flavonoids and polyphenols (Bouarab-Chibane et al., 2019, Fujimoto and Masuda, 2012, Górniak et al., 2019, Xie et al., 2015).

The biofilm forming ability of probiotics plays an important role in the successful colonization and the effectiveness of the treatment of dysbiosis. This property allows them to withstand the environmental conditions, leading to the colonization and sustainability of their population (Salas-Jara et al., 2016). The five tested probiotics in this study could be divided into two categories: non-biofilm formers (Protexin and Enterol) and weak biofilm formers (BioGaia, Linex and Normaflore). Application of PEE treatment caused various effects on the weak biofilm forming probiotics. The weak biofilms of BioGaia and Linex microbes were suppressed by low concentrations of PEE (12.5 and 25 µg/ml, respectively), while in case of Normaflore only the highest concentration (800 µg/ml) was able to inhibit the biofilm formation. The antibiofilm properties of PEE could be related to its flavonoids and polyphenols content. In addition to their destructive activity on bacteria, flavonoids and polyphenols can suppress the biofilm formation process by altering bacterial adhesion, motility (Górniak et al., 2019) and the regulatory mechanisms of the bacterial population such as quorum sensing or other universal regulator systems (Slobodníková et al., 2016). However, the interesting effect of low concentrations of PEE on the biofilm of Normaflore microbes, where the biofilm was enhanced and shifted from weak to strong, might be attributable to the enhancement of autoaggregation (Sorroche et al., 2012) and/or the inhibition of swarming motility (O’May et al., 2012). The results of autoaggregation test showed positive correlation between autoaggregation and biofilm forming ability of Normaflore microbes. Similar direct relationship has been reported by Sorroche et al. (2012) in *Sinorhizobium meliloti*. According to their suggestions, the same physical adhesive forces are responsible for both biofilm forming ability and autoaggregation (Sorroche et al., 2012). On the other hand, PEE treatment inhibited the swarming motility of Normaflore microbes indicating an inverse relationship with biofilm forming ability. Similar inverse relationship was documented by several previous studies (Chakroun et al., 2018, O’May et al., 2016, O’May et al., 2012). It is assumed that once the bacteria start the attachment in the biofilm formation process, and due to the PEE-induced autoaggregation and the inhibition of surface-associated swarming motility, the attachment of more planktonic cells will lead to the formation of microcolonies, that can later lead to the formation of mature biofilm (Caiazza et al., 2007, O’May and Tufenkji, 2011).

With respect to biofilm eradication experiment, Normaflore microbes were not only able to resist PEE, but even the biofilm status was shifted from weak to strong at low concentrations (12.5–25 µg/ml). This considerable increment in the biofilm mass might be due to the prebiotic effect of PEE. Moreover, it is proposed that the proliferation of the planktonic cells released from the mature biofilm, with enhanced autoaggregation at low concentrations of PEE, allows more cells to be reintroduced and increase the biofilm mass. At higher concentrations (50–400 µg/ml), where the planktonic cells were no more viable, only the prebiotic effect of PEE was responsible for the increment of the biofilm mass. However, only the highest concentration of PEE (800 µg/ml) was able to eradicate the biofilm mass. The eradication process may occur due to the disturbance of bacterial adhesion and quorum sensing (Górniak et al., 2019), the interaction with the extrapolymeric substances of the biofilm, and killing mechanism of bacteria inside the biofilm, leading to detachment of the biofilm from the substratum (Chen et al., 2018).

## 5 Conclusion

In conclusion, the results of this study revealed that PEE has concentration and strain dependent effect on the viability and biofilm forming ability of the probiotics *in vitro*. Propolis, in certain cases, can act as prebiotic at low concentrations, however, at higher concentrations it may inhibit the planktonic growth and biofilm forming ability of the probiotics. Therefore, more attention should be paid for the selection of the appropriate probiotics used for the treatment of dysbiosis, and on the other hand for the simultaneous application of PEE. The present observations showed limitations for the co-application of PEE and probiotics and adumbrated a potential double-face action of PEE on the natural gut microbiota or pathogenic microorganisms.

## Funding

This research did not receive any specific grant from funding agencies in the public, commercial, or not-for-profit sectors.

## Declaration of Competing Interest

The authors declare that they have no known competing financial interests or personal relationships that could have appeared to influence the work reported in this paper.
